# Lymphatic filariasis control in Tanzania: effect of six rounds of mass drug administration with ivermectin and albendazole on infection and transmission

**DOI:** 10.1186/1471-2334-13-335

**Published:** 2013-07-21

**Authors:** Paul E Simonsen, Yahya A Derua, William N Kisinza, Stephen M Magesa, Mwele N Malecela, Erling M Pedersen

**Affiliations:** 1 DBL – Centre for Health Research and Development, Faculty of Health and Medical Sciences, University of Copenhagen, Thorvaldsensvej 57, 1871 Frederiksberg C, Denmark; 2 Amani Medical Research Centre, National Institute for Medical Research, P.O. Box 81, Muheza, Tanzania; 3 RTI International, International Development Group, Nairobi, Kenya; 4 National Institute for Medical Research, P.O. Box 9653, Dar es Salaam, Tanzania

## Abstract

**Background:**

Control of lymphatic filariasis (LF) in most countries of sub-Saharan Africa is based on annual mass drug administration (MDA) with a combination of ivermectin and albendazole, in order to interrupt transmission. We present findings from a detailed study on the effect of six rounds of MDA with this drug combination as implemented by the National Lymphatic Filariasis Elimination Programme (NLFEP) in a highly endemic rural area of north-eastern Tanzania.

**Methods:**

The effect of treatment on transmission and human infection was monitored in a community- and a school-based study during an 8-year period (one pre-intervention and 7 post-intervention years) from 2003 to 2011.

**Results:**

Before intervention, 24.5% of the community population had microfilariae (mf) in the blood, 53.3% had circulating filarial antigens (CFA) and 78.9% had specific antibodies to the recombinant filarial antigen Bm14. One year after the sixth MDA, these values had decreased considerably to 2.7%, 19.6% and 27.5%, respectively. During the same period, the CFA prevalence among new intakes of Standard 1 pupils in 10 primary schools decreased from 25.2% to 5.6%. In line with this, transmission by the three vectors (*Anopheles gambiae*, *An. funestus* and *Culex quinquefasciatus*) as determined by dissection declined sharply (overall vector infectivity rate by 99.3% and mean monthly transmission potential by 99.2% between pre-intervention and fifth post-intervention period). A major shift in vector species composition, from predominantly anopheline to almost exclusively culicine was observed over the years. This may be largely unrelated to the MDAs but may have important implications for the epidemiology of LF in the area.

**Conclusions:**

Six MDAs caused considerable decrease in all the measured indices for transmission and human infection. In spite of this, indices were still relatively high in the late period of the study, and it may take a long time to reach the recommended cut-off levels for interruption of transmission unless extra efforts are made. These should include increased engagement of the target population in the control activities, to ensure higher treatment coverage. It is expected that the recent initiative to distribute insecticide impregnated bed nets to every household in the area will also contribute towards reaching the goal of successful LF elimination.

## Background

Lymphatic filariasis (LF), a disfiguring and disabling disease caused by a mosquito-borne parasitic infection, is a major public health problem in many developing countries with a warm and humid climate. The causative nematode parasite in sub-Saharan Africa is *Wuchereria bancrofti*, and it has been estimated that more than 45 million people are affected in this region [1]. The parasites are transmitted to humans when infected mosquito vectors deposit infective larvae onto the human skin [2]. The larvae penetrate the skin, migrate to the lymphatic vessels, and develop into male and female adult worms over a period of months. Mature and fertilized female worms release large numbers of minute microfilariae (mf) which circulate in the blood. Mf ingested by a vector during a blood meal will develop to infective larvae in about 10-14 days. These migrate to the mosquito’s proboscis and may then be transmitted to a new human host during a subsequent blood meal. The mosquito vectors thus play an essential role in maintaining the life cycle and disseminating the infection. Clinical disease primarily results from damage caused by the adult worms in the lymphatic vessels. The common clinical manifestations (e.g. acute filarial fever, lymphoedema, elephantiasis, hydrocele) can incur considerable incapacity to the affected individuals, with consequent loss of income and social and psychological stress, and LF has been recognized a leading cause of long-term disability in the world [3].

A large Global Programme to Eliminate Lymphatic Filariasis (GPELF), launched in 2000 by the World Health Organization, has targeted LF for elimination [4]. The GPELF provides guidance and support to national control programmes. The principal intervention measure recommended by GPELF is annual mass drug administration (MDA) of two-drug combinations to LF endemic communities. In most endemic countries a combination of diethylcarbamazine (DEC) and albendazole is used, but due to the risk of serious adverse reactions in individuals infected with *Onchocerca volvulus*, a combination of ivermectin and albendazole is used in African countries which are co-endemic for onchocerciasis. The drugs are primarily microfilaricidal and the MDAs rarely completely clear the *W. bancrofti* infection from the treated individuals. However, it is the strategy that the reduction in microfilarial load in the endemic population will lead to a simultaneous reduction of transmission, and that the MDAs thereby will prevent new infections to establish. With time the already established infections will also die out and LF will be eliminated. The term “transmission control” has been adopted for this strategy.

In Tanzania, with an estimated 34 million people at risk and 6 million people affected [5], a National Lymphatic Filariasis Elimination Programme (NLFEP) was established and began operations in 2000. The main strategy of the programme is to apply annual MDAs with a combination of ivermectin (150-200 μg/kg) and albendazole (400 mg) to individuals aged 5 years and above in selected programme areas. Tanga Region, located in the north-eastern part of Tanzania bordering the Indian Ocean, was enrolled in the NLFEP, and endemic areas received the first MDA, in October 2004. A study to monitor the effect of the programme was initiated before the onset of the MDAs in order to obtain baseline data on infection and transmission and to subsequently monitor the effect of control. We have previously reported from the early part of this study, namely from a community-based part carried out in the highly endemic village of Kirare [6], and from a school-based part assessing the effect in young pupils from 10 rural primary schools [7]. Here we report on the effect of six rounds of MDA from both the community-based and the school-based part of the study.

## Methods

### Study sites

**Figure 1:**
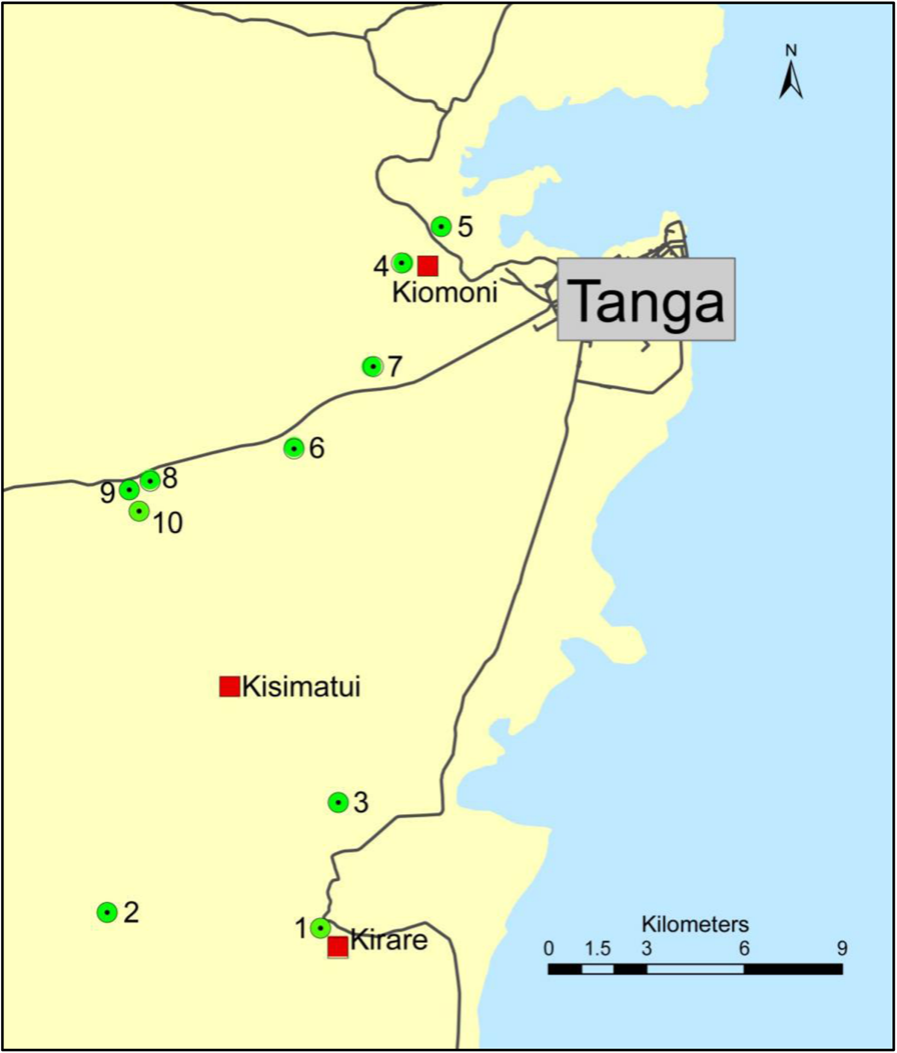
**Map of study area showing location of the three villages (Kirare, Kiomoni and Kisimatui) and ten schools.** Schools: 1. Kirare, 2. Mapojoni, 3. Marungu, 4. Kiomoni, 5. Mafuriko, 6. Maweni, 7. Kange, 8. Pongwe, 9. Kigandini, and 10. Ziwani.

All study sites were located in rural areas of Tanga District in Tanga Region, north-eastern Tanzania. The study had two parts, a community based and a school based (Figure 1). The community part was primarily carried out in Kirare village (5°15’01” S, 39°01’40” E) located about 20 km south of Tanga city along the Tanga–Pangani road. In 2010, two other villages were included, namely Kiomoni (5°04’01” S, 39°03’17” E) located about 5 km north-west of Tanga city near the Tanga-Amboni road, and Kisimatui (5°11’0” S, 39°0’0” E) located about 17 km south-west of Tanga city along the Pongwe-Marungu road. The school part was carried out in ten rural primary schools located to the south-west (Kirare, Mapojoni and Marungu; no. 1-3), north-west (Kiomoni and Mafuriko; no. 4-5), close west (Maweni and Kange; no. 6-7) and more distant west (Pongwe, Kigandini and Ziwani; no. 8-10) of Tanga city. The distance from the schools to the centre of Tanga city ranged from 5 to 24 km.

### MDA for LF control in the study area

The Tanzanian National Lymphatic Filariasis Elimination Programme (NLFEP) administered the first three rounds of MDA in Tanga District in October 2004, February 2006 and May 2007, as reported [6]. This was followed by three additional rounds in February 2009, November 2009 and December 2010 (Table 1). The six MDAs were thus administered with time intervals of 16, 15, 21, 9 and 13 months.

**Table 1:** Timing of mass drug administration (MDA) and major village and school survey activities

**MDA**	**Village surveys**	**School surveys**
	Sep-04: Survey 1, in hamlets 1, 2, 3 and 4 (mf, clin, Og4C3, Bm14)	Sep-04: Survey 1 (ICT)
Oct-04: MDA1		
	Jan-06: Survey 2, in hamlets 1, 2, 3 and 4 (mf, Og4C3, Bm14)	Sep-05: Survey 2 (ICT)
Feb-06: MDA2		
	Jan-07: Survey 3, in hamlets 1, 2, 3 and 4 (mf, Og4C3, Bm14)	Oct-06: Survey 3 (ICT)
May-07: MDA3		
	Oct-07: Survey 4, in hamlets 1, 2, 3 and 4 (mf, Og4C3, Bm14)	Oct-07: Survey 4 (ICT)
	Oct-08: Survey 5, in hamlets 1, 2, 3 and 4 (mf, Og4C3, Bm14)	Oct-08: Survey 5 (ICT)
Feb-09: MDA4		
	Oct-09: Survey 6, in hamlets 1, 2, 3 and 4 (mf)	Oct-09: Survey 6 (ICT)
Nov-09: MDA5		
	Nov-10: Survey 7, in hamlets 1, 2, 5 and 6 (ICT, mf)	Nov-10: Survey 7 (ICT, Bm14)
Dec-10: MDA6		
	Nov-11: Survey 8, in hamlets 1, 2, 5 and 6 (ICT, mf, Bm14)	Dec-11: Survey 8 (ICT, Bm14)

Hamlets 1, 2, 3 and 4 were in Kirare village, hamlet 5 in Kiomoni village and hamlet 6 in Kisimatui village. Types of examinations: mf = microfilariae; clin = chronic clinical manifestations; Og4C3 = circulating filarial antigens by TropBio Og4C3 ELISA; ICT = circulating filarial antigens by ICT cards; Bm14 = antibodies to recombinant Bm14 antigen (by in-house ELISA during village surveys 1-5; by CELISA from Cellabs during surveys 7 and 8). In addition to listed activities, entomological surveillance was carried out in Kirare village continuously, and questionnaire surveys for MDA coverage was carried out in all communities and schools shortly after each MDA (the post-MDA 5 and 6 questionnaires in communities also assessed for possession of bed nets). A population census covering hamlets included in subsequent surveys was done in 2004 (before Survey 1) and 2010 (before Survey 7). Impregnated bed nets were distributed to every household in the study area in September 2011.

### Design of community study

Entomological surveillance started in Kirare in November 2003, i.e. about one year before the first MDA, and continued uninterrupted until the end of the present reporting period.

Cross sectional community surveys for filarial infection (microfilaraemia, circulating filarial antigenemia and antibodies to Bm14) and clinical manifestations were carried out in Kirare immediately before the first MDA (September 2004; survey 1). It was intended to make similar follow-up surveys for infection shortly before each of the following MDAs, but due to irregularities in timing of the MDAs this plan could sometimes not be adhered to (Table 1). Follow-up surveys were thus carried out in January 2006, January 2007, October 2007, October 2008, October 2009, November 2010 and November 2011 (survey 2-8). Surveys 1-6 were based on a census made by the project in 2004 (updated for survey 6 by removing dead and immigrated individuals). These surveys were carried out as previously described [6], i.e. all individuals were examined for mf during night-time and individuals living in the 50 mosquito collection houses had a venous blood sample taken for determination of circulating filarial antigenemia and antibodies to Bm14 by ELISA (no venous samples in survey 6).

In the following two surveys (7 and 8) several changes were introduced (Table 1). First, only two of the four hamlets comprising Kirare village (Mtambuuni and Mashine) were included, as growing study-fatigue was faced in the other two hamlets (Korosini and Tundaua). As substitutes, and in order to also cover a larger geographical area, one hamlet from each of two other rural villages in Tanga District (Mabavu hamlet in Kiomoni village, and Majengo hamlet in Kisimatui village), which each had approximately the same population size as the remaining part of Kirare, were included. Second, a new census was made in all three villages in 2010 as a basis for the surveys. Third, instead of screening all individuals for mf during night-time, all individuals were first screened for CFA with rapid test cards during the day, and only those positive for CFA were screened for mf at night. Fourth, no venous blood samples were collected. Instead, blood spots were collected on filter paper disks during survey 8 (for later screening for antibodies to Bm14) from children aged 5-14 years immediately after the CFA testing.

### Design of school study

The new Standard 1 pupils from ten selected primary schools were examined for CFA in the late part of each year, as previously described [7]. On agreed days and by following the school registers, the pupils were examined for CFA by use of rapid test cards. In 2010 and 2011, finger-prick blood was moreover collected on filter paper disks immediately thereafter (in Kirare, Marungu, Kiomoni and Mafuriko schools only), for later screening for antibodies to Bm14 (Table 1).

### Ethical considerations

Meetings were held regularly in the study communities to inform the inhabitants about the study contents and findings and to obtain their cooperation. Prior to any examination, the individuals were asked if the purpose and consequences as explained during the meetings had been understood, questions for clarification were answered, and written consent to participate (from adults, and from parents or guardians of individuals less than 15 years old) was obtained.

Prior to each years’ school surveys, meetings were held with the head teacher, the relevant teachers and the parents committee for the individual schools, to explain the contents and consequences of the study. The members of the parents committee provided written informed consent, on behalf on the children and their parents. Children who refused to participate in the examinations were not included.

Ethical and research clearance for the study was provided by the Medical Research Coordinating Committee of the National Institute for Medical Research, Tanzania, and the study protocol was reviewed by the Central Scientific Ethical Committee in Denmark.

### Test for microfilariae

Blood was examined for mf by use of the counting chamber technique [8]. Sampling started at 21.00 hours. From each individual, 100 μl of finger prick blood was collected in a heparinized capillary tube and transferred to a tube with 900 μl of 3% acetic acid. Later in the laboratory, specimens were transferred to a counting chamber and examined for mf under a compound microscope. The specimens were examined blindly by two different technicians, and the mean count was recorded as the mf intensity.

### Tests for circulating filarial antigens and antibodies to Bm14

During community surveys 1-5, venous blood samples were collected from volunteers from the mosquito collection houses in Kirare, and serum was prepared and examined for circulating filarial antigens (CFA) by the TropBio Og4C3 ELISA and for antibodies to the recombinant filarial antigen Bm14 by an in-house ELISA as previously reported [6].

During community surveys 7-8, and during all school surveys, individuals were screened for CFA by use of rapid immunochromatographic test cards (ICT cards, Binax Now®, Inverness Medical Innovations Inc., USA). One hundred microliters of finger-prick blood were applied to the sample pad on the test card, and the result was read after exactly 10 minutes as either positive or negative.

During community survey 8, and during school surveys 7 and 8, finger-prick blood was moreover collected on filter paper collection disks (TropBio Pty. Ltd., Townsville, Australia) from some individuals for later detection of antibodies to Bm14. Each protrusion on the disk was saturated with blood from one individual. The disks were dried overnight, placed individually in plastic bags and frozen at -20°C until use. Elution of the dried blood samples and subsequent determination of antibodies to Bm14 was done by use of the Filariasis CELISA test kit (Cellabs Pty Ltd, Brookvale, Australia). The test was performed according to the procedure from the manufacturer and as described elsewhere [9,10]. Each sample was tested in duplicate and the mean OD-value was recorded as the individuals’ result. Individuals with OD-values ≥ 0.40 were considered antibody positive.

### Entomological surveillance

Vector mosquitoes were collected from 50 originally randomly selected village houses using Centre for Disease Control light traps (John W. Hock Company, Gainesville, USA) hung beside an occupied, untreated bed-net [6]. A few houses were changed over the years, either because inhabitants refused continued trapping in their house or because they moved away from the village and left the house to collapse, and in these cases a neighbouring house was used instead. Traps were switched on at 1900 hours and off at 0600 hours by trained field assistants. Caught mosquitoes were transferred to paper cups and transported to the laboratory in Tanga for identification using morphological criteria. The live female vectors (*An. gambiae*, *An. funestus* and *Cx. quinquefasciatus*) were dissected under microscope for larvae of *W. bancrofti*.

### Assessment of treatment coverage and bed net use

Three different methods were used to assess the treatment coverage. First, the official programme coverage for Tanga District was obtained from the regional office of the NLFEP in Tanga. According to the programme, these “reported coverages” were calculated as the number of treatments delivered divided by the eligible population (estimated as 80% of the total population). In addition, a “surveyed community coverage” and a “surveyed school coverage” was obtained from questionnaire surveys carried out among the inhabitants from the study communities and the Standard 1 pupils from the study schools, respectively, shortly after each MDA. In these surveys, individuals were asked in privacy whether or not they had taken the drugs that had been distributed for LF elimination (in communities, parents answered on behalf of their children below 15 years of age).

Only few houses in Kirare possessed bed nets at the start of the study in 2003, but nets were observed to gradually become more common. During the post-MDA 5 and 6 community questionnaire surveys for MDA coverage, individuals aged ≥ 15 years were therefore also asked about the number and type of bed nets possessed in their household (was checked by visual inspection when inhabitants allowed), and the number of individuals ≥ 1 year sleeping in the household. After MDA 5, net coverages were 27.6% (366 nets/1326 individuals) for any kind of net and 6.6% (87/1326) for insecticide treated nets in Kirare (all four hamlets). After MDA 6, these net coverages were 27.0% (452/1674) and 7.1% (118/1674), respectively, for the study hamlets of Kirare, Kiomoni and Kisimatui combined. Insecticide impregnated bed nets were distributed to every household in Tanga District in September 2011, shortly before completion of the present study.

### Data analysis

Mf intensities were adjusted for sampling time by multiplying with a time-specific factor, as previously described [11]. Geometric mean intensities (GMIs) of microfilaraemia, CFA intensities and Bm14 OD-values were calculated as antilog [(Σlog x + 1)/n]-1, with x being the mf/ml, CFA units or OD-values, respectively, and n the number of individuals examined.

During community surveys 7 and 8, when only CFA positive individuals were examined for mf, the community mf prevalence was calculated as: (b/a) × (d/c) × 100, where a = number of individuals in the community examined for CFA, b = number of those examined for CFA being positive, c = number of CFA positives examined for mf, and d = number of those examined for mf being positive. In these surveys, the community mf GMI was calculated as antilog [(Σlog x + 1)/(c/b × a)]-1, with x being the mf/ml and (c/b × a) being the number of individuals examined (taking into account that sometimes not all CFA positive individuals were examined for mf).

Entomological indices for vector biting and transmission were calculated as previously described [12]. Briefly, the monthly biting rate (MBR; a measure of the number of mosquito bites per person in the month) was calculated as: (total mosquito catch × days in month × 3)/(number of catching nights × number of light traps × 2). The monthly transmission potential (MTP; a measure of the number of infective larvae to which a person is exposed in the month) was calculated as: (MBR × total number of infective larvae seen in the dissections)/(number of mosquitoes dissected). The “infectivity rate” was calculated as the percent of mosquitoes infected with infective larvae (L3) and the “infection rate” as the percent of mosquitoes infected with any stage of the parasite (L1, L2 and/or L3).

## Results

### Lymphatic filariasis in Kirare before treatment

Table 2 gives an overview of the LF infection and disease status in the human population of Kirare as seen during the pre-MDA survey in September 2004. The population was heavily affected, with community prevalence rates of 24.5%, 53.3% and 78.9% for mf, CFA and filarial (Bm14) specific antibodies, respectively. 4.1% of the adults (≥ 20 years) had elephantiasis and 32.8% of the adult males had hydrocele. Statistical comparison of the LF status in the overall population of Kirare (all four hamlets) to that in the village section included in the later part of the study (Mtambuuni and Mashine hamlets only) indicated no significant differences.

**Table 2:** Overview of the LF status in Kirare, as seen in the pre-MDA survey in September 2004

**Characteristic**	**Kirare village (all 4 hamlets)**	**Mtambuuni and Mashine hamlets**	**p-value (*χ***^**2**^**-test)**
Registered population ≥ 1 yr	1112	530	-
Examined population ≥ 1 yr	919	471	-
Male : female ratio among examined	0.88	0.85	0.76
Proportion of examined below 20 yrs	53.0%	48.4%	0.11
Mf prevalence	24.5%	26.1%	0.51
Mf GMI* among all examined	4.11	4.83	-
Mf GMI* among mf positive	781.3	851.1	-
CFA prevalence^#^	53.3%	54.7%	0.87
Bm14 prevalence^#^	78.9%	78.1%	0.91
Hydrocele prevalence (in males ≥ 20 yrs)^§^	35.4%	45.2%	0.11
Elephantiasis prevalence (in all ≥ 20 yrs)^§^	4.2%	5.8%	0.35

Shown for the complete village (Mtambuuni, Korosini, Mashine and Tundaua hamlets) and for the village section included in the later part of the study (Mtambuuni and Mashine hamlets only).

* Geometric mean intensity, in mf/ml blood.

^#^ Based on volunteers from mosquito collection houses only (90 and 64 examined individuals from the four and two hamlets of Kirare, respectively).

^§^ Assessed in 175 and 104 males (hydrocele), and in 431 and 242 males and females (elephantiasis), in the four and two hamlets of Kirare, respectively.

**Figure 2:**
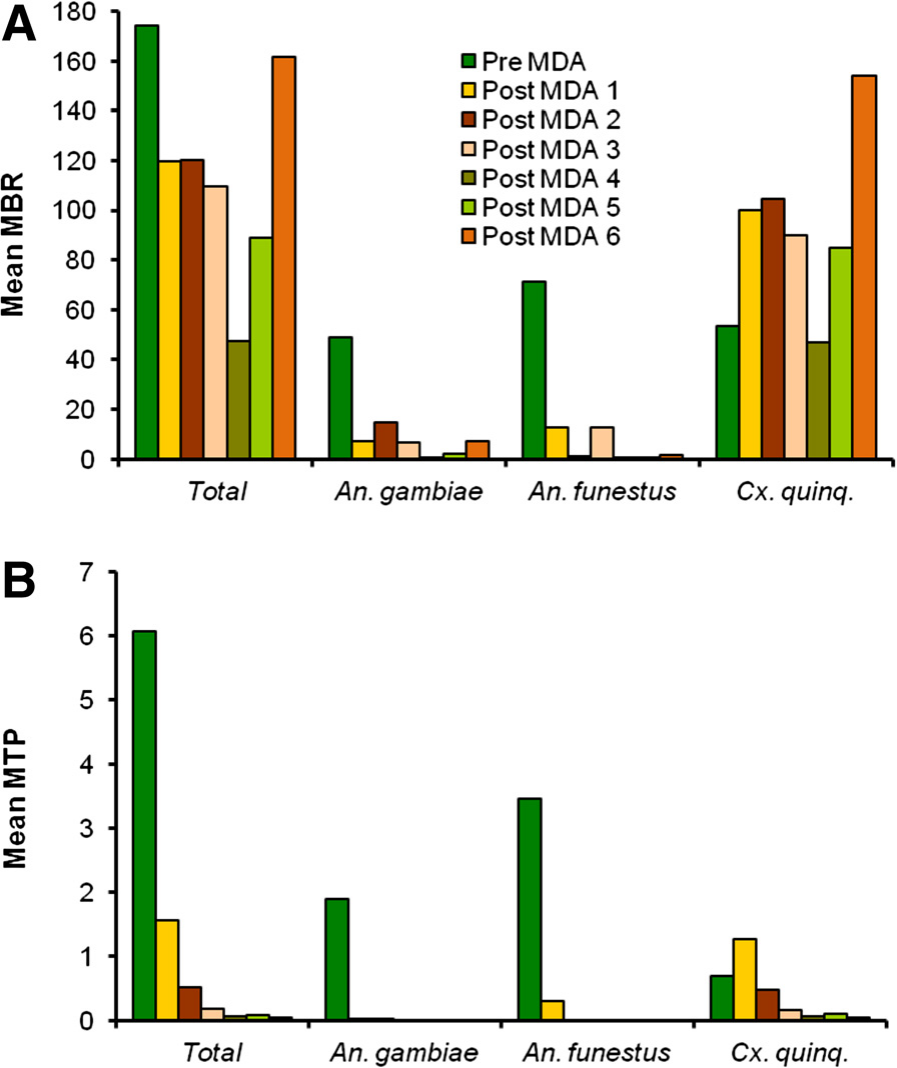
**Vector-biting and transmission in the pre-MDA period and the six post-MDA periods in Kirare. A** shows mean monthly biting rate (MBR) and **B** shows mean monthly transmission potential (MTP).

**Figure 3:**
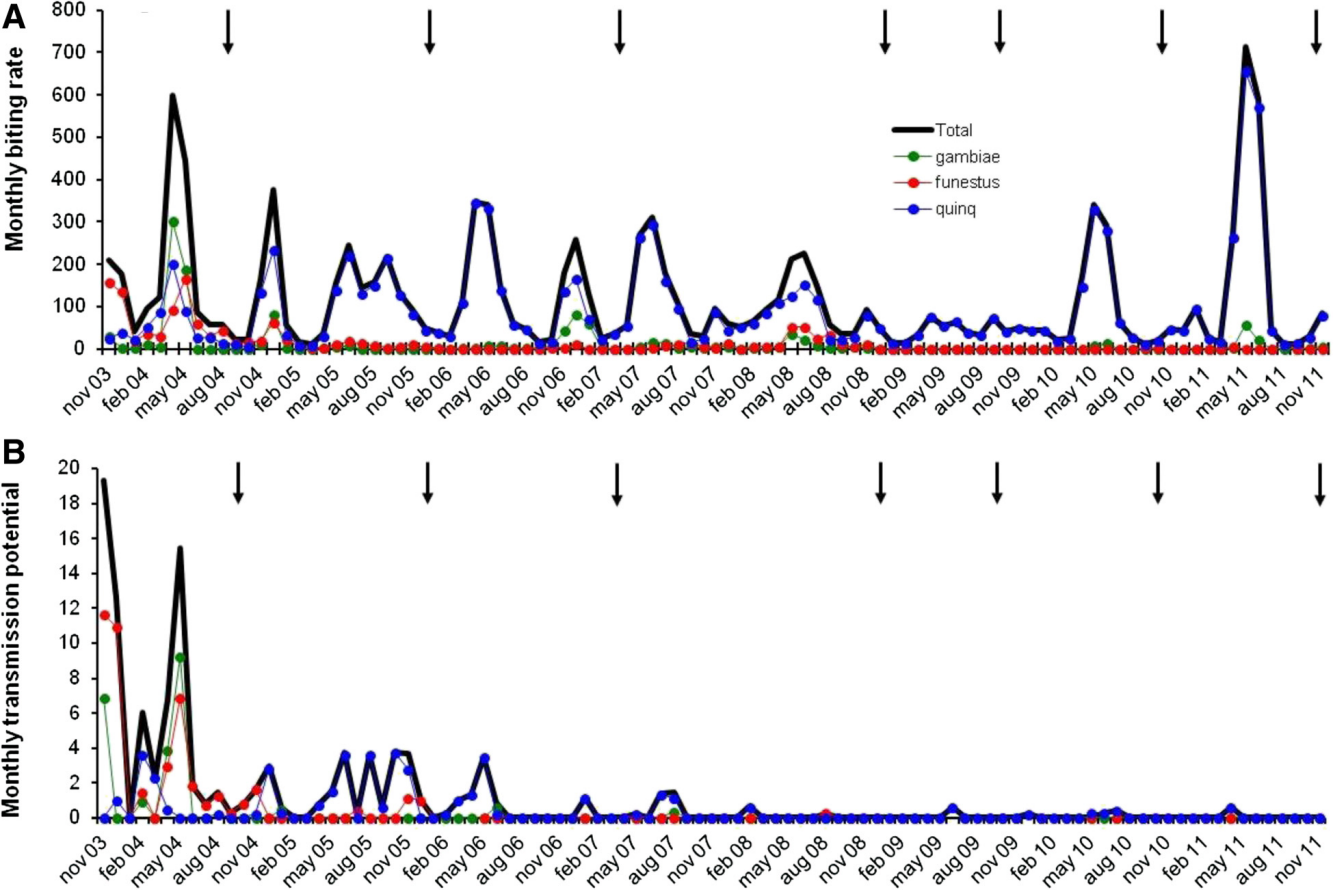
**Seasonal variation in monthly vector-biting and transmission in Kirare (November 2003 to November 2011). A** shows monthly biting rate (MBR) and **B** shows monthly transmission potential (MTP). Thick black line = all vector species combined; Thin green line = *Anopheles gambiae*; Thin red line = *An. funestus*; Thin blue line = *Culex quinquefasciatus*; Arrows indicate rounds of mass drug administration.

Dissection of mosquitoes caught from the 50 collection houses during the 11 months pre-MDA period indicated that the area had three species of vectors (Table 3), and that these had a combined mean MBR of 174.3 and mean MTP of 6.1 (Figure 2). *An. funestus* was the most abundant (mean MBR of 71.3) and contributed most to transmission (mean MTP of 3.5) followed by *An. gambiae* (49.3 and 1.9, respectively) and *Cx. quinquefasciatus* (53.6 and 0.7, respectively). Vector biting and transmission varied by season (Figure 3), and generally was most pronounced during and after the rainy seasons (November-December and April-June).

**Table 3:** Vector mosquito catches from the 50 collection houses in Kirare and the outcome of dissections

	**Total**	***An. gambiae***	***An. funestus***	***Cx. quinquefasciatus***
Pre-MDA period (11 months)*				
No. mosquitoes caught	8346	2335	3385	2626
No. mosquitoes dissected	5396	1477	2080	1839
No. with infection (% of dissected^#^)	187 (3.47)	56 (3.79)	94 (4.52)	37 (2.01)
No. with L3 (% of dissected^§^)	77 (1.43)	20 (1.35)	48 (2.31)	9 (0.49)
No. of L3	153	51	87	15
Post-MDA period 4 (9 months)**				
No. mosquitoes caught	1930	7	5	1918
No. mosquitoes dissected	1830	5	4	1821
No. with infection (% of dissected^#^)	6 (0.33)	0 (0.00)	0 (0.00)	6 (0.33)
No. with L3 (% of dissected^§^)	1 (0.05)	0 (0.00)	0 (0.00)	1 (0.05)
No. of L3	3	0	0	3
Post-MDA period 5 (13 months)***				
No. mosquitoes caught	5148	119	27	5002
No. mosquitoes dissected	3976	71	23	3882
No. with infection (% of dissected^#^)	9 (0.23)	0 (0.00)	0 (0.00)	9 (0.23)
No. with L3 (% of dissected^§^)	4 (0.10)	0 (0.00)	0 (0.00)	4 (0.10)
No. of L3	4	0	0	5
Post-MDA period 6 (12 months)****				
No. mosquitoes caught	8982	416	74	8492
No. mosquitoes dissected	7713	387	74	7252
No. with infection (% of dissected^#^)	2 (0.03)	0 (0.00)	0 (0.00)	2 (0.03)
No. with L3 (% of dissected^§^)	1 (0.01)	0 (0.00)	0 (0.00)	1 (0.01)
No. of L3	2	0	0	2

Results shown are from the pre-MDA period and post-MDA periods 4-6. Results from post-MDA periods 1-3 are given in [6].

* Nov-03 to Sep-04; ** Feb-09 to Oct-09; *** Nov-09 to Nov-10; **** Dec-10 to Nov-11.

^#^ Infection rate.

^§^ Infectivity rate.

### Effect of MDAs on mf in community surveys

**Figure 4:**
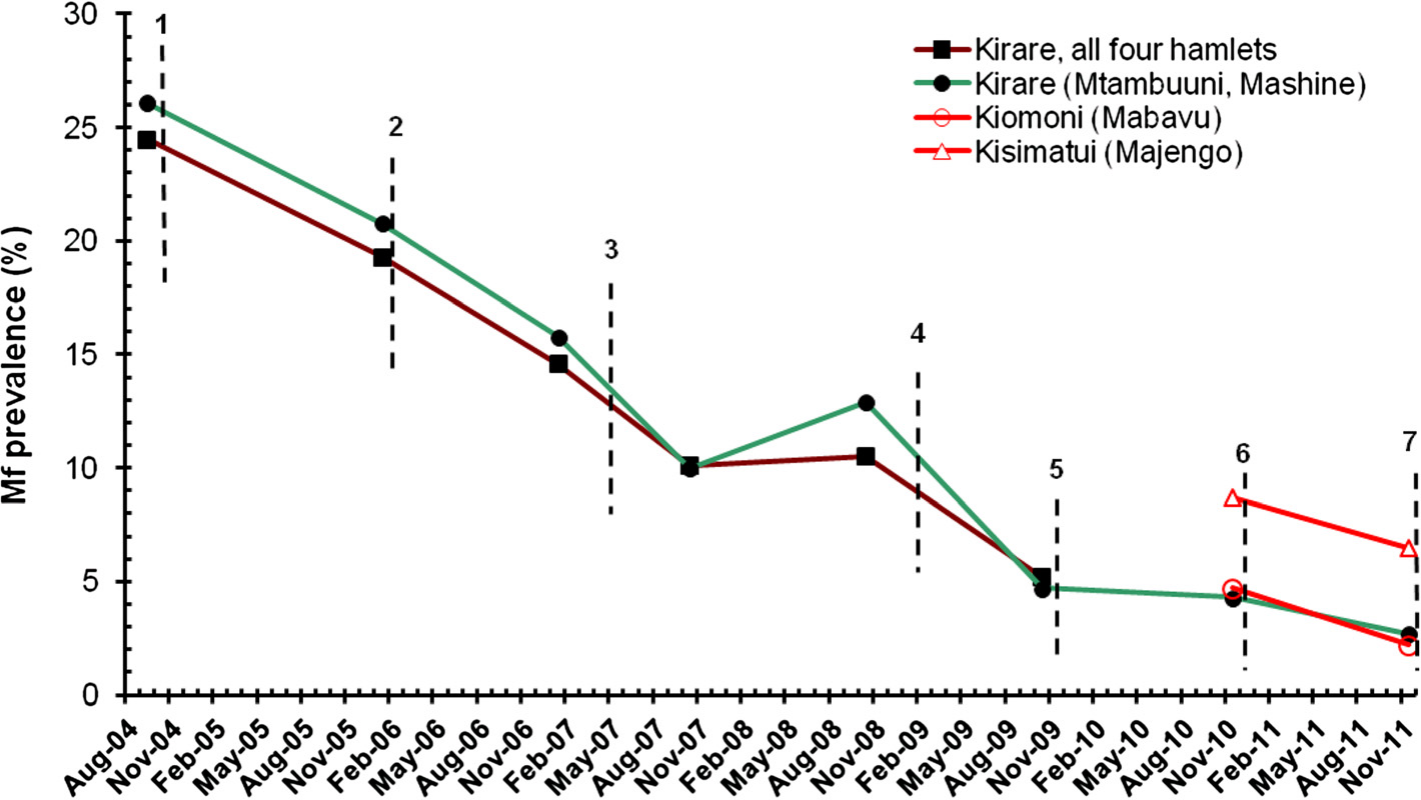
**Effect of six rounds of mass drug administration (MDA) on *Wuchereria bancrofti *microfilaria prevalence.** Brown line = all four hamlets of Kirare in survey 1-6; Green line = two hamlets of Kirare (Mtambuuni, Mashine) in survey 1-8; Red lines = one hamlet of Kiomoni (Mabavu) and one hamlet of Kisimatui (Majengo) in survey 7 and 8. In survey 1-6 all individuals ≥ 1 year were examined for mf. In survey 7 and 8 all individuals ≥ 1 year were first examined for CFA, and those positive were examined for mf. Vertical stippled lines indicate rounds of MDA.

In the early part of the study (survey 1-6), all individuals in Kirare were examined for mf. The mf prevalence and mf GMI for all 4 hamlets combined in survey 1-5 were presented previously [6]. Following an initial steep and statistically significant decrease in mf prevalence and mf GMI after each of the first three MDAs there was a levelling out of the effect between survey 4 and 5, probably because of the extended delay of MDA 4. In survey 6 (survey population: 1029; number examined: 477), the mf prevalence and mf GMI decreased to 5.5% and 0.34 mf/ml (from 10.6% and 0.75 mf/ml during survey 5), respectively. When tested for individuals included in both surveys these decreases were statistically significant (n = 262; McNemar’s chi-square test for paired samples, p = 0.02 and paired *t*-test, p = 0.03, respectively). Thus, there was a general trend of a continued and statistically significant decrease in microfilaraemia as long as the spacing of MDAs did not become too long. The prevalence for all hamlets of Kirare combined in survey 1-6 are shown in Figure 4.

When looking at the two hamlets of Kirare which were also examined in the later part of the study (Table 4 and Figure 4), the mf prevalence and mf GMI during the first six surveys followed the trend seen for all four hamlets combined. Thus, after each of the first three MDAs the mf prevalence decreased progressively and statistically significantly (n = 421, 395 and 329; p < 0.001, p = 0.01 and p < 0.001, McNemar’s test), from 26.1% in the pre-MDA survey to 10.0% in survey 4. This was followed by a non-significant increase to 12.9% in survey 5 (probably due to the prolonged period without treatment) and a significant decrease to 5.0% in survey 6 (n = 254 and 160; p = 0.19 and p = 0.013, McNemar’s test). The mf prevalence continued its downward move to 4.4% and 2.7% in survey 7 and 8, respectively (statistics cannot be computed due to the method used for assessing mf prevalence in these surveys, see Methods and Table 4). The community mf GMI followed a similar trend, with significant decrease after each MDA from pre-MDA survey to survey 4, then a slight but non-significant increase in survey 5, followed by a significant decrease in survey 6 (p < 0.001, p < 0.001, p = 0.005, p = 0.13 and p = 0.039; paired *t*-test). In the following two surveys the community mf GMI continued to decrease to 0.25 and 0.16 mf/ml, respectively (again, statistics cannot be computed). When considered for mf positive individuals only, the mf GMIs initially decreased considerably from pre-MDA survey to survey 3, but thereafter remained relatively high for the remaining study period, probably due to systematic non-compliance to treatment of some mf positive individuals.

**Table 4:** Microfilaraemia in the study communities

**Study population**	**Survey no.**	**Time of survey**	**Survey population**	**No. examined (%)**	**No. positive for mf (%)**	Mf GMI* for all examined	Mf GMI* for positives
Kirare	1	Sep-04	530	471 (88.9)	123 (26.1)	4.83	851.1
	2	Jan-06	530	461 (87.0)	96 (20.8)	2.46	385.7
	3	Jan-07	530	438 (82.6)	69 (15.8)	1.08	101.5
	4	Oct-07	530	351 (66.2)	35 (10.0)	0.70	200.3
	5	Oct-08	530	302 (57.0)	39 (12.9)	0.90	142.4
	6	Oct-09	512	259 (53.5)	13 (5.0)	0.30	194.7
	7	Nov-10	690	400 (58.0)^#^	17^§^ (4.4)	0.25^¤^	178.9
	8	Nov-11	690	393 (57.0)^#^	11^§^ (2.7)	0.15^¤^	180.8
Kiomoni	7	Nov-10	504	386 (76.6)^#^	18^§^ (4.7)	0.34^¤^	423.1
	8	Nov-11	504	312 (61.9)^#^	7^§^ (2.2)	0.16^¤^	487.3
Kisimatui	7	Nov-10	651	389 (59.8)^#^	34^§^ (8.7)	0.55^¤^	159.1
	8	Nov-11	651	367 (56.4)^#^	24^§^ (6.5)	0.33^¤^	77.2

Results shown are for two hamlets of Kirare (Mtambuuni, Mashine) in survey 1-8, as well as for one hamlet of Kiomoni (Mabavu) and one hamlet of Kisimatui (Majengo) in survey 7 and 8. Results from all four hamlets of Kirare in survey 1-5 are given in [6].

* Geometric mean intensity, in mf/ml blood.

^#^ Number (%) examined for CFA. In survey 7 and 8, the “number of CFA positives/number of CFA positives examined for mf/number of mf positives detected” were 101/89/15 and 77/65/9 for Kirare, 74/68/17 and 52/52/7 for Kiomoni and 110/91/28 and 84/63/18 for Kisimatui, respectively.

^§^ Expected number of mf positives among those examined for CFA. Calculated as [number positive for CFA] × [number positive for mf] / [number CFA positives examined for mf].

^¤^ Expected mf GMI among those examined for CFA. See Methods for calculation.

The mf prevalence and mf GMI in Kiomoni and Kisimatui (Table 4, Figure 4) also followed a downward trend between survey 7 and 8 (statistics cannot be computed). In both surveys, these indices were considerably higher in Kisimatui than in Kiomoni and Kirare.

### Effect of MDAs on CFA and antibodies to Bm14 in community surveys

**Figure 5:**
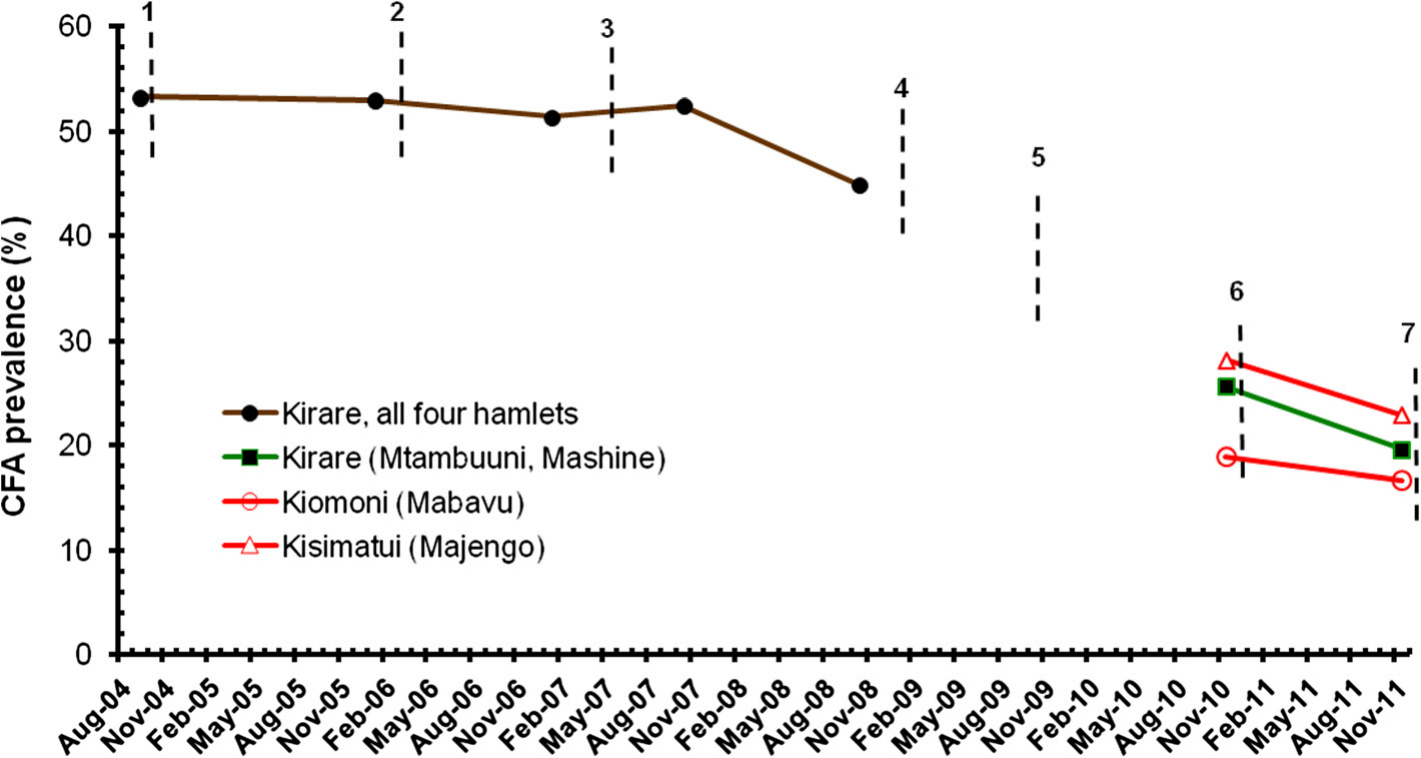
**Effect of six rounds of mass drug administration (MDA) on circulating filarial antigen (CFA) prevalence.** Brown line = volunteers from mosquito collection houses located in all four hamlets of Kirare examined for CFA by ELISA in survey 1-5. Green line = all individuals ≥ 1 year from two hamlets of Kirare (Mtambuuni, Mashine) examined for CFA by ICT cards in survey 7 and 8. Red lines = all individuals ≥ 1 year from one hamlet of Kiomoni (Mabavu) and one hamlet of Kisimatui (Majengo) examined for CFA by ICT cards in survey 7 and 8. Vertical stippled lines indicate rounds of MDA.

In the early part of the study (survey 1-5), the effect of MDAs on CFA and antibodies to Bm14 was assessed by ELISA on serum samples from volunteers from the 50 mosquito collection houses in Kirare (see details in [6]). For CFA, a slow decrease was seen in prevalence from 53.3% in survey 1 to 44.9% in survey 5 (Figure 5), whereas a more substantial decrease was seen for the GMIs (reduction by 56%). For antibodies to Bm14, a negligible decrease in prevalence was seen from 78.9% in survey 1 to 75.5% in survey 5, whereas again a more substantial decrease was seen for the GMIs (reduction by 48%).

In survey 7 and 8, all individuals in the study communities were examined for CFA with ICT cards (Table 5; Figure 5). The prevalence in Kirare had decreased considerably from the 44.9% in survey 5 (all four hamlets) to 25.3% and 19.6% in survey 7 and 8 (two hamlets only), respectively. In all three villages of Kirare, Kiomoni and Kisimatui, the decrease in CFA prevalence between survey 7 and 8 was statistically significant (Table 5).

**Table 5:** Circulating filarial antigen (CFA) prevalence in the study communities

**Study population**	**Time (number) of survey**	**Survey population**	**No. examined (%)**	**No. positive (%)**	**p-value for difference in prevalence**
Kirare	Nov-10 (7)	690	400 (58.0)	101 (25.3)	0.044*
	Nov-11 (8)	690	393 (57.0)	77 (19.6)	
Kiomoni	Nov-10 (7)	504	386 (76.6)	74 (19.2)	0.041*
	Nov-11 (8)	504	312 (61.9)	52 (16.7)	
Kisimatui	Nov-10 (7)	651	389 (59.8)	110 (28.3)	0.001*
	Nov-11 (8)	651	367 (56.4)	84 (22.9)	
All 3 communities combined	Nov-10 (7)	1845	1175 (63.7)	285 (24.3)	< 0.001^#^
	Nov-11 (8)	1845	1072 (58.1)	213 (19.9)	

Results shown are from two hamlets of Kirare (Mtambuuni, Mashine), one hamlet of Kiomoni (Mabavu) and one hamlet of Kisimatui (Majengo) in survey 7 and 8. Results from all four hamlets of Kirare in survey 1-5 are given in [6] and in Figure 3.

* McNemar’s chi-square test for paired samples (n = 318, 289, 297 and 904 for Kirare, Kiomoni, Kisimatui and all three villages combined, respectively).

^#^ Pearsons chi-square test.

In survey 8, children aged 5-14 years from Kirare, Kiomoni and Kisimatui were examined for antibodies to Bm14 by CELISA (Table 6). The prevalence in Kirare had decreased considerably from the 75.5% in survey 5 (all four hamlets, all age groups) to 27.5% in survey 8 (two hamlets, children only), and the GMI of the CELISA OD-value was reduced by 16% (from 0.405 to 0.338) between these surveys. Similar levels of prevalence and OD-value GMI were seen in the three villages in survey 8 (mean of 23.0% and 0.2865, respectively).

**Table 6:** Antibodies to Bm14 in children from the study communities and the study schools

	**Study population**	**Time (number) of survey**	**Survey population**	**No. examined (%)**	**Mean age years**	**No. positive (%)**	GMI* for all examined
Community surveys	Kirare	Nov-11 (8)	178	120 (67.4)	10.1	33 (27.5)	0.3384
	Kiomoni	Nov-11 (8)	145	107 (73.8)	9.9	24 (22.4)	0.2776
	Kisimatui	Nov-11 (8)	180	129 (71.7)	9.4	25 (19.4)	0.2468
	All 3 communities combined	Nov-11 (8)	503	356 (70.8)	9.8	82 (23.0)	0.2865
School surveys	All 4 schools combined	Nov-10 (7)	453	362 (79.9)	7.4	61 (16.9)^#^	0.2776^§^
		Dec-11 (8)	382	334 (87.3)	7.2	38 (11.4)^#^	0.2153^§^

Results shown are from children (5-14 years) from two hamlets of Kirare (Mtambuuni, Mashine), one hamlet of Kiomoni (Mabavu) and one hamlet of Kisimatui (Majengo) in community survey 8, and from Standard 1 children from four of the study schools (Kirare, Marungu, Kiomoni and Mafuriko) in survey 7 and 8. Findings from survey 1-5 in Kirare (all four hamlets) are given in [6].

* Geometric mean intensity, in OD-values.

^#^ p = 0.039 (Pearsons chi-square test).

^§^ p = 0.032 (independent sample *t*-test on log-transformed OD-values).

### Effect of MDAs on vectors and transmission in Kirare

A considerable change in vector species composition was observed already in the early study period [6], from predominantly anopheline in the pre-MDA period (*An. gambiae* and *An. funestus* comprising 68.5% of the catch) to predominantly culicine in the post-MDA 3 period (*Cx. quinquefasciatus* comprising 82.0% of the combined catch; p < 0.001, Pearson chi-square test). This change became even more pronounced in post-MDA period 4-6, when *Cx. quinquefasciatus* comprised 95.6% of the combined catch (Table 3; p < 0.001, Pearsons chi-square test comparing post MDA period 1-3 to post MDA period 4-6). The overall mean MBR was 174.3 in the pre-MDA period (Figure 2). It decreased gradually to 47.4 in post-MDA period 4, but thereafter increased again to 89.1 and 161.8 in post-MDA period 5 and 6, respectively, due to an increase in the culicine population. In addition to these long-term changes, there were marked seasonal fluctuations in vector species composition and abundance (Figure 3A).

When analysed for the three vector species combined, the vector infectivity rate decreased markedly and statistically significantly in the early part of the study, from 1.43% in the pre-MDA period to 0.10% in the post-MDA 3 period (p < 0.001, chi-square test; see also [6]). The vector infectivity rate decreased further after subsequent MDAs, to 0.05% and 0.01% in post-MDA periods 4 and 6 (Table 3), respectively, although changes from post-MDA 3 period and onwards did not reach statistical significance due to the low number of infective vectors relative to the number of vectors dissected. The downward trend in infectivity rate was seen for all three vector species. However, whereas no infective larvae were seen in the two anopheline species in post-MDA period 4, 5 and 6 (very few anophelines were caught), infective *Cx. quinquefasciatus* were recovered in all periods (Figure 3B, Table 3). The decrease in vector infectivity rates resulted in progressively less *W. bancrofti* transmission (Figure 2). Thus, the overall mean MTP decreased from 6.08 in the pre-MDA period to 1.56, 0.53, 0.19, 0.07, 0.08 and 0.05 in post-MDA period 2, 3, 4, 5 and 6, respectively (reductions by 74.3%, 91.3%, 96.9%, 98.8%, 98.7% and 99.2%, respectively, in relation to the pre-MDA value). Meanwhile *Cx. quinquefasciatus* gradually became responsible for more and more of the transmission, and in the last three post-MDA periods this species was the only observed vector.

### Effect of MDA in school surveys

**Figure 6:**
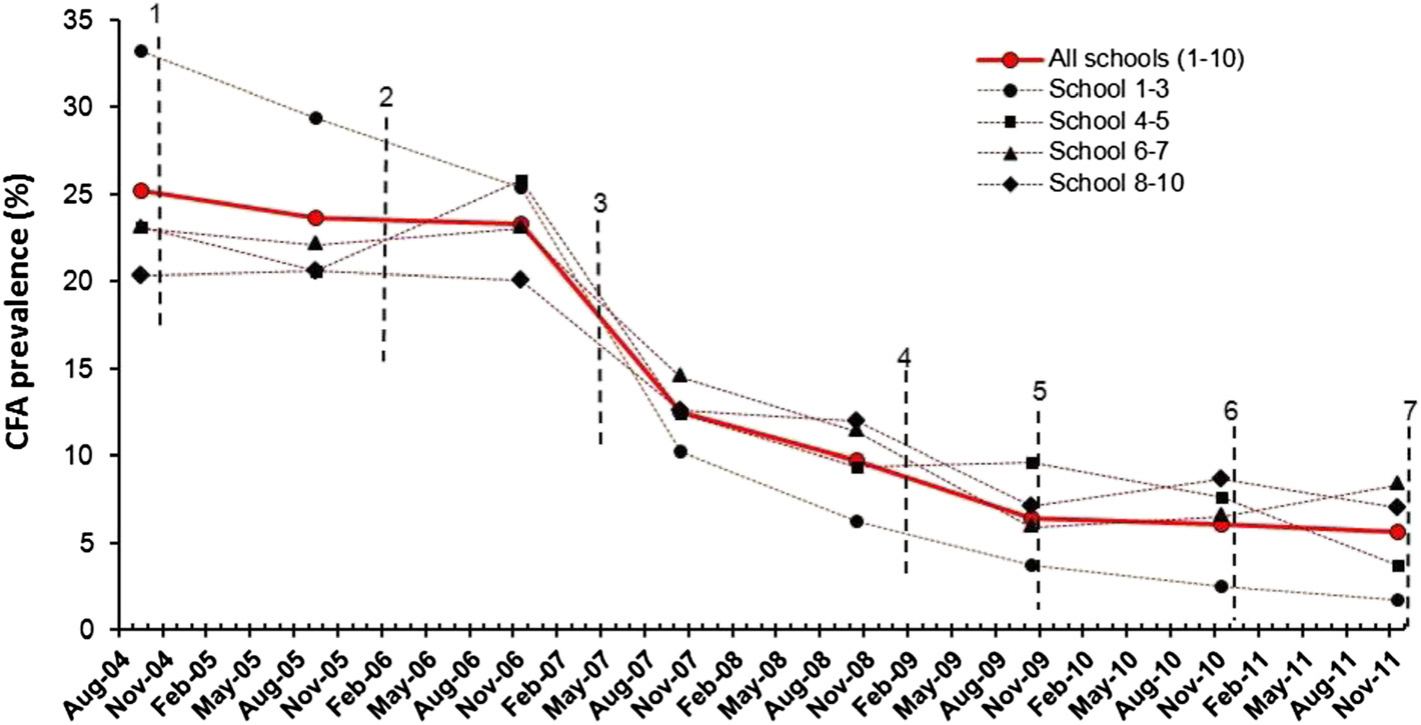
**Effect of six rounds of mass drug administration (MDA) on circulating filarial antigen (CFA) prevalence in Standard 1 children.** Thick line = prevalence for all 10 schools combined. Thin stippled lines = prevalence in school clusters according to their location to the south-west (Kirare, Mapojoni and Marungu schools; no. 1-3), north-west (Kiomoni and Mafuriko schools; no. 4-5), close west (Maweni and Kange schools; no. 6-7) and more distant west (Pongwe, Kigandini and Ziwani schools; no. 8-10) of Tanga. Vertical stippled lines indicate rounds of MDA.

Approximately 800 new Standard 1 pupils from the 10 study schools were examined each year shortly after enrolling in school. The overall CFA prevalence was 25.2% in the pre-MDA survey (Table 7; Figure 6). The prevalence showed only minor and non-significant change to 23.6% and 23.3% in survey 2 and 3, respectively. This was followed by more substantial and statistically significant decreases to 12.5%, 9.7% and 6.4% during the next three surveys (see also [7]). The decrease thereafter levelled off, and prevalences reached 6.1% and 5.6% in survey 7 and 8. When comparing these prevalences to those seen one year earlier, or comparing the prevalence in survey 6 to that in survey 8, the differences were not statistically significant (p = 0.80, 0.65 and 0.48, respectively, Pearsons chi-square test). Similar trends of CFA prevalence decrease were seen in the four school clusters (Figure 6).

**Table 7:** Circulating filarial antigen (CFA) prevalence in Standard 1 pupils from the 10 primary schools

**Time (number) of survey**	**No. pupils registered**	**No. examined (% of registered)**	No. girls/boys (ratio)*	Mean age in years (range)*	**No. positive for CFA (% of examined)**	**p-value for change in CFA prevalence from previous year**^**#**^
Sep-04 (1)	888	832 (93.7)	399/433 (0.92)	8.1 (6-14)	210 (25.2)	-
Sep-05 (2)	892	691 (77.5)	337/354 (0.95)	7.5 (6-14)	163 (23.6)	0.50
Oct-06 (3)	981	803 (81.9)	406/397 (1.02)	7.9 (6-12)	187 (23.3)	0.84
Oct-07 (4)	953	806 (84.6)	433/373 (1.16)	7.7 (6-16)	101 (12.5)	< 0.001
Oct-08 (5)	971	848 (87.4)	419/429 (0.98)	7.6 (6-12)	82 (9.7)	0.064
Oct-09 (6)	855	760 (88.9)	386/374 (1.03)	7.6 (6-12)	49 (6.4)	0.018
Nov-10 (7)	966	831 (86.0)	404/427 (0.95)	7.5 (6-12)	51 (6.1)	0.80
Dec-11 (8)	943	889 (94.3)	421/468 (0.92)	7.5 (6-11)	50 (5.6)	0.65

Each year, the new intake of Standard 1 pupils in the 10 schools (Kiomoni, Mafuriko, Marungu, Kirare, Mapojoni, Pongwe, Kigandini, Maweni, Ziwani and Kange) was examined for CFA with ICT cards.

* For examined.

^#^ Pearsons chi-square test.

The Standard 1 pupils from four of the schools (Kirare, Marungu, Kiomoni and Mafuriko) were moreover examined for antibodies to Bm14 in survey 7 and 8 (Table 6). Despite the minor change in CFA prevalence between these two surveys, the Bm14 antibody response decreased significantly, both when considering prevalence of positivity (from 16.9% to 11.4%, p = 0.026; Pearsons chi-square test) and GMI of OD-values (from 0.2776 to 0.2153, p = 0.032, *t*-test). The slightly lower values observed in children in the school surveys compared to those in the communities (Table 6) are likely to be due to the lower mean age of the former than the later group (7.2 vs. 9.8 years in 2011).

### MDA treatment coverage

The treatment coverage in MDA 1-6, as assessed by three different methods, is shown in Table 8. The official NLFEP “reported coverage” for Tanga District was very high for all MDAs, with three of the treatment rounds covering 95% or more of the eligible population. The “surveyed community coverage” obtained from interviews in the study communities was generally lower than the “reported coverage” by 7-30% (with exception of MDA 1). The “surveyed school coverage” obtained from interviews with the Standard 1 pupils from the 10 study schools was even lower (again with exception of MDA 1), in particular during the last three MDAs where only half or less of the children reported that they had taken the treatment.

**Table 8:** Assessment of treatment coverage during mass drug administration (MDA) 1-6

**MDA no.**	**Time of MDA**	Reported coverage for the district in%*	**Surveyed coverage in%**	
			**Community study**^**#**^	**School study**^**§**^
1	Oct-04	80.0	82.3	80.0
2	Feb-06	95.0	79.0	68.2
3	May-07	87.0	69.9	61.2
4	Feb-09	96.6	66.8	32.9
5	Nov-09	98.7	86.4	43.1
6	Dec-10	75.0	67.9	50.3

* Official NLFEP coverage for eligible individuals in Tanga District, where the three study villages and ten study schools are located.

^#^ Assessed by interviews shortly after each MDA in the study communities (975, 1009, 883, 930, 778 and 1462 individuals aged ≥ 5 years interviewed after MDA 1-6, respectively).

^§^ Assessed by interviews shortly after each MDA in the 10 study schools (859, 676, 776, 836, 704 and 746 Standard 1 pupils interviewed after MDA 1-6, respectively).

## Discussion

Following the launching of GPELF in 2000 and the targeting of LF for global elimination, most endemic countries have established national control programmes and many are in the process of implementing annual MDAs with the recommended two-drug combinations [4]. Regular monitoring of the effect of the programmes is essential to evaluate the progress, to make evidence based adjustments and to ultimately end the MDAs when specified programme stopping criteria have been met [13,14]. Thorough assessments and analyses of the effect of MDAs on transmission and human infection in sentinel sites have been documented, especially from countries using the DEC/albendazole combination such as Egypt [15], Papua New Guinea [16,17], American Samoa [18,19], India [20] and Samoa [21]. Many LF endemic countries in sub-Saharan Africa are co-endemic for onchocerciasis, and due to the potential risk of DEC induced side-effects in individuals with *Onchocerca volvulus* infections, use a combination of ivermectin and albendazole in their MDAs for LF control. The effect of this drug combination in a low-moderate LF endemic area of central Nigeria, West Africa, was recently documented [22,23]. Here we present and analyse the effect of six rounds of MDA with ivermectin and albendazole in a highly endemic area of Tanzania, East Africa.

The coastal area of north-eastern Tanzania is well known to be endemic for LF, and has been a focus for comprehensive research on epidemiology and measures for control of LF in the past e.g. [24-30]. Prior to start-up of the NLFEP-activities in Tanga Region in 2004, no control had taken place in any of the sites used in the present study. In consistency with this, it was noted that pre-MDA indices of LF infection and transmission were very high and resembled those reported during earlier studies in nearby areas. High levels of clinical manifestations were also recorded in Kirare study community, and were moreover strikingly visible when moving around in the area.

The study design and methodology and the effects of MDAs in the early study period was reported previously for Kirare [6] and the 10 schools [7]. Some changes in study design and methodology in the community study were necessitated in later study period. First, increasing study fatigue among inhabitants of Kirare resulted in decreasing survey coverage from one MDA to the next. This was not only due to the yearly blood sampling, but also to some political rivalry among groups of villagers. As a consequence, it was decided to leave out two of the village hamlets and instead focus more attention on the remaining two hamlets where most cooperation was met. To compensate for the smaller survey population in Kirare, two other communities were included, whereby also a larger geographical area was covered. Second, due to the reduced human infection burden after the first MDAs there was a need to adopt a new and more practical diagnostic approach. The primary community screening tool was therefore changed from mf-testing to CFA-testing by ICT cards [31], followed by examination of only CFA positive individuals for mf. As it was still essential to have an idea about the mf prevalence and intensity in the community (as indicators of availability of infection to the vectors), methods for calculating these indices based on the new screening procedure were developed. Third, the availability of a new commercial kit for examination of dried finger prick blood spots on filter paper eliminated the need for venous blood sampling in the later part of the study and made it practically feasible to examine larger populations for antibodies to Bm14. The changes in methods for measuring both CFA and antibodies to Bm14 are not likely to have had a major impact on the obtained results, but should still be kept in mind when analysing and interpreting the findings. In contrast to the opposition sometimes experienced from villagers during the human surveys, the trapping of mosquitoes in the 50 houses of Kirare generally proceeded smoothly throughout the study period.

In Kirare, the mf prevalence and community mf GMI generally decreased progressively with increasing number of MDAs. An exception to this was survey 5, where slight but statistically insignificant increases in these indices were seen, most likely due to the long interval between MDA 3 and 4 (21 months). An increase in mf and CFA was reported from Haiti after a single MDA with DEC/albendazole had been missed [32], and both of these observations therefore seem to emphasize the importance of keeping a relatively short spacing between the MDAs. Decreases in mf prevalence and community mf GMIs were also obvious between survey 7 and 8 in the two new communities. Mf GMIs calculated on the basis of mf positive individuals generally remained high throughout the study, suggesting that some mf positive individuals regularly either avoided or were left out from treatment during the MDAs (systematic non-compliance). These individuals comprise an important source of mf for the continued transmission of LF.

CFAs are primarily released by adult *W. bancrofti*, and their presence is a sensitive and specific indirect measure of adult worm infection [16]. In the early study period, when CFAs were measured by ELISA, the decrease in CFA prevalence in Kirare was small. A more substantial decrease was seen in the GMIs, most likely because intensities were extremely high in the early period of the study. Thus, although intensities decreased they only reached the cut-off level for positivity in a few individuals [33]. In the late study period, when CFAs were measured with ICT cards, prevalences were substantially lower, and a continuing and statistically significant decrease was seen between survey 7 and 8 in all three communities. The two tests for CFA detection (ELISA and ICT cards) have been shown to be well in agreement when measuring CFA status [34]. Although the community CFA prevalence decreased considerably from the pre-MDA survey to survey 8, it was still quite high after 6 MDAs.

The specific antibody response to the filarial antigen Bm14 is used as an indirect marker of exposure to *W. bancrofti* transmission [21,35]. In the early part of the study the Bm14 antibodies were measured with ELISA (by using antigen donated by Prof. G. Weil through the NIH/NIAID Filariasis Research Repository Centre, Smith College, USA) and all age-groups were examined, whereas in the later part of the study tests were performed with a commercially available test-kit (with antigen from the same source) and only included children aged 5-14 years. Although the different techniques used and age groups examined prevent exact comparisons, the very pronounced decrease in both prevalence and OD-value GMI between the pre-MDA survey and survey 8 in Kirare indicated that a major decrease in exposure to transmission had taken place in the human population, which is also in alignment with the findings from the entomological surveillance.

The vector species composition changed considerably during the study period from predominantly anopheline during the pre-MDA period, to almost exclusively culicine in the late study period. This remarkable shift has been documented and analysed in more detail elsewhere [36,37]. With respect to the role of the MDAs, there is documented evidence that anopheles mosquitoes taking a blood meal on ivermectin treated individuals show increased mortality [38], and that MDAs for LF in this way may affect transmission of malaria [39]. It is, however, difficult immediately to accept that the MDAs should be a primary cause for the shift, and a multitude of factors, including those related to environmental and climate change may be involved. Whatever the reason, there can be no doubt that the shift has important consequences for the epidemiology and control of LF, as the anopheline and culicine vector species differ in biology, biting habits and vectorial capacity. In addition to the shift in species composition there were marked seasonal fluctuations in vector abundance, with most vectors being present during and shortly after the two annual rainy seasons. Transmission was also most intense, and during the late period of the study only observed, in these seasons.

Transmission, assessed by dissection of vectors and recovery of L3s, decreased after each MDA, from high levels in the pre-MDA period to very low levels in the post MDA 6 period. It is likely that most of the decrease in transmission was due to the MDAs, which reduced the availability of microfilariae in the human population to the vectors. However, part of the decrease may also be due to the shift in vector species composition, whereby long-lived strongly anthropophilic anophelines (and thereby highly efficient vectors) were replaced by shorter lived and less strongly anthropophilic culicines (and thereby less efficient vectors) [12]. In the later period of the study, only very few infective mosquitoes were identified despite large numbers of vectors being dissected. Collection and dissection of vectors is a time-consuming and expensive task, but it provides the most exact information about vector dynamics and transmission of LF and as such is an important research tool for detailed investigation of transmission [40]. Newly developed PCR techniques may replace the laborious dissection activity in future assessments. However, the number of mosquitoes to be collected, and the efforts to be invested in collecting them, in order to get statistical reliable information about change in transmission intensity in the late period of control programmes may be enormous. In the present study, where mosquitoes were collected one night each week from 50 houses by use of light traps, too few were obtained to demonstrate a statistical change in transmission after post MDA 3 (although a decreasing trend was seen). Under these conditions, either much more effective mosquito collection methods are needed or indirect assessment methods such as measurement of CFA or antibodies to Bm14 in the human population (especially children) have to be employed.

The school based study was used as an alternative approach to assess the effect of MDAs on transmission in the area. The idea being that a reduced transmission will lead to reduced acquisition of infection in young children, and thereby a reduction in prevalence of CFA when examining children of the same age from year to year after start of MDA [7]. Indeed, after the first three MDAs the CFA prevalence in the new intake of Standard 1 pupils decreased rapidly, and thus provided a strong indirect indication that children were less and less exposed to infection from year to year after start of MDA. Further decrease in CFA prevalence was noted in the last two surveys, but the effect levelled off and the prevalence was still 5.6% in survey 8. The majority of children examined during the last two surveys were aged one year or less when the first MDA was administered in 2004 (67.7% in survey 7 and 90.7% in survey 8) and among these the CFA prevalence (5.9% in survey 7 and 5.1% in survey 8) was comparable to the overall CFA prevalence for these surveys. Examination of the Standard 1 pupils from four of the schools for Bm14 antibody in survey 7 and 8 showed relatively high rates of positivity. However, the significant reduction in both Bm14 prevalence and OD-value GMI between these two surveys suggests that transmission was on the decrease.

Sustained high drug-intake coverage during MDAs for LF control is critical in order to reach the programme target within a reasonable time-frame, in particular in areas like the present with very high pre-MDA levels of infection [41,42]. Treatment coverage and compliance are therefore important factors to consider when monitoring and assessing the impact of MDAs, but reliable information is difficult to get. In the present study we used three different approaches to assess treatment coverage, namely the official “reported coverage” from NLFEP for Tanga District and two “surveyed coverages” obtained from interviews carried out shortly after each MDA in the study communities (individuals aged 5 years and above) and the study schools (Standard 1 pupils), respectively. Apart from MDA 1, where all three approaches indicated about 80%, the surveyed coverages were considerably lower than the reported coverage, in particular for the Standard 1 pupils. The discrepancy in treatment coverage obtained by different approaches in this area, and the possible courses, was recently addressed in detail in a social science study [43], and only few comments related specifically to observations at the present study sites will be added. Firstly, there were frequent complaints from inhabitants in the study communities that they were not well informed by the programme about the drug distributions beforehand, neither regarding timing or purpose. Second, it was noted by the research team that the method for drug distribution varied between different MDAs and different sites, and obviously house-to-house distribution was more effective than distribution from a central village point. The very low coverages among the Standard 1 children, especially during the last three MDAs were noted to be related to: drug distribution during school holidays, some schools had no distribution point nearby, and parents refusing treatment of their children due to rumours of side effects. Finally, the activities of the research project probably created extra awareness about LF and its control in the study communities, which may have resulted in higher treatment coverages during MDAs than in other communities in the area. These important issues should be addressed in subsequent treatment rounds, in order to ensure sufficient treatment coverage, and thereby effectiveness, of the MDAs.

## Conclusions

The present study demonstrated that, when compared to the pre-MDA scenario, six MDAs resulted in considerable decrease in infection and transmission of LF in the study populations, whether these were communities or schools and whether measures were based on detection of mf, CFA, antibodies to Bm14 or mosquito vector dissections. In spite of this, there is still some way to go before the recommended target cut-off level of 1% CFA for interrupting transmission and stopping MDA [14] is reached, and it may take a long time unless extra efforts are made. These should particularly include strengthened information dissemination to - and engagement of - the target populations in control activities, to ensure higher treatment coverages. The drawbacks that may be caused by much delayed MDAs should be avoided, and perhaps even more frequent treatments may be necessary in the last phase of the programme. Environmental management to limit breeding of the *Cx. quinquefasciatus* vectors should also be encouraged. It is likely that the recent initiative to distribute insecticide impregnated bed nets to every household in the area will contribute substantially to further transmission reduction [41,42], but also for this measure to show its full potential the importance of thoroughly informing and engaging the target communities should not be underestimated. Monitoring and evaluation will continue to play an important role to guide the programme and to ensure that the current major achievements will ultimately lead towards the goal of successful LF elimination.

## Competing interests

The authors declare that they have no competing interests.

## Authors’ contributions

PES, YAD, WK, SMM, MNM and EMP conceived and designed the experiments. PES, YAD, SMM and EMP performed the experiments. PES, YAD and EMP analysed the data. PES, YAD and EMP wrote the paper. All authors read and approved the final manuscript.

## Pre-publication history

The pre-publication history for this paper can be accessed here:

http://www.biomedcentral.com/1471-2334/13/335/prepub
